# Chronic spontaneous urticaria: a low-grade disseminated intravascular coagulation only partially reversed by Omalizumab

**DOI:** 10.1007/s10238-022-00838-9

**Published:** 2022-05-25

**Authors:** Marina Di Pino, Maria Filomena Ruberto, Giulia Costanzo, Davide Firinu, Maria Sebastiana Piras, Mario Nicola Mura, Stefano Del Giacco, Ferdinando Coghe, Francesco Marongiu, Doris Barcellona

**Affiliations:** Department of Medical Sciences and Public Health, and Unit of Internal Medicine, University of Cagliari, Policlinico Universitario – AOU di Cagliari Azienda Ospedaliero Universitaria, SS 554-Bivio Sestu, 09042 Monserrato, CA Italy

**Keywords:** Chronic spontaneous urticaria, Omalizumab treatment, Clot waveform analysis, Endogenous thrombin generation, D-Dimer

## Abstract

Chronic spontaneous urticaria (CSU) is a disorder characterized by wheals and/or angioedema. The coagulation cascade and inflammation pathways are closely linked together. The aim of our study was first to investigate the dynamics of clot formation in plasma (Clot Waveform Analysis, CWA) in a group of 47 patients with CSU along with other coagulative parameters dedicated to the study of hypercoagulability, such as D-Dimer, F 1 + 2 peptide, Fibrinogen, Platelet count and Mean Platelet Volume (MPV). Secondly, 23 out of 47 patients were treated with Omalizumab at four administration intervals from T0 to T4. A statistically significant increase in Activated Partial Thromboplastin (aPTT) ratio, D-Dimer, F1 + 2, Platelet count and MPV was found when compared with 53 healthy controls (HC). In contrast, the 2nd Derivative of aPTT showed lower values than those of the HC. No differences were found between 1st derivative of aPTT and Fibrinogen. D-Dimer only showed a significant difference between T0 and T3. An activation of both coagulation and fibrinolysis along with a weaker clot acceleration may be in agreement with a low-grade DIC. The accelerated turnover of platelets expressed by both an increase in platelet count and MPV further supports this pathway in CSU. Omalizumab does not affect the relationship between the immune and the hemostatic systems.

## Introduction

Chronic spontaneous urticaria (CSU) is a common disorder characterized by spontaneous onset of itchy wheals and/or angioedema, lasting for six or more weeks [1]. It could be debilitating, difficult to treat, and frustrating for patients and physicians [2]. Despite the effort to obtain an effective therapy for CSU, a disease-modifying drug is not yet available. A large part of patients may experience symptoms relapse of CSU after a variable withdrawal from treatment.

The role of the coagulation cascade in the CSU physiopathology is known but poorly understood. It has been ascertained that the coagulation cascade and inflammation pathways are closely linked together [3]. A proper stimulus on eosinophils can act as a trigger for tissue factor expression which activates the extrinsic pathway of coagulation cascade [4].

The aim of our study was first to investigate the dynamics of clot formation in plasma (Clot Waveform Analysis, CWA) in a group of 47 patients with CSU along with other coagulative parameters dedicated to the study of hypercoagulability, such as D-Dimer, F 1 + 2 peptide, Fibrinogen, Platelet count and Mean Platelet Volume (MPV) in comparison with a group of healthy subjects (HC). Secondly, 23 out of 47 patients were treated with Omalizumab at four administration intervals. Omalizumab is a monoclonal antibody (mAb) directed to the Ce3 domain of human IgE heavy chain, the site that binds to Fc receptors on mast cells and basophils [5]. Omalizumab has been approved for the treatment of CSU by the regulatory agencies. Its favorable efficacy and safety have been confirmed by several meta-analysis [6–8]. In this subgroup of patients treated with Omalizumab we added the measurement of the Thrombin Generation to the other tests described above.

Then, we divided the patient group into two subgroups: CSU on treatment and CSU on anti H1 treatment (CIA).

## Patients and methods

A total of 47 patients with CSU (8 males and 39 females, median age 44 years, range 14–71 years), from outpatient unit of the Internal Medicine Service, Allergology and Clinical Immunology clinics of the Teaching Hospital “Duilio Casula,” A.O.U. of Cagliari, from December 2017 to November 2019, were enrolled in this study. A group of 53 HC (16 males and 37 females, median age 43 years, range 16–70 years) was also studied.

In a total of 23 out of 47 patients (4 males and 19 females, median age 43 years, range 19–70 years, median UAS7: 28, range 19–42) we collected plasma samples on a scheduled planning:

- T0 before first Omalizumab infusion (300 mg subcutaneously, every 4 weeks)
- T1 after 4 weeks as second Omalizumab infusion
- T2 after 8 weeks as third Omalizumab infusion
- T3 after 24 weeks as sixth Omalizumab infusion
- T4 after 28 weeks as 4 weeks after the last Omalizumab infusion.

They fulfilled the inclusion criteria:

- Diagnosis of CSU lasting 6 weeks or more, based on “The EAACI/GA^2^LEN/EDF/WAO guidelines for the definition, classification, diagnosis and management of urticarial” [1].
- On-going therapy with anti H1 at the maximum dose
- UAS7 score > 35 at the first visit.

We did not include patients previously treated with Omalizumab or cyclosporin, younger than 16 years old or having known cardiovascular diseases or active cancer.

We collected for each patient demographic and clinical data. (Table 1).

**Table 1:** Demographic and clinical data in 47 CSU patients at T0 (before first Omalizumab infusion)

Demographic and clinical characteristics at T0	Median, range
Years from diagnosis	6.00, 2.00–21.00
IgE total	172.00, 8.00–2131.00
Eosinophils (× 10^3^ /µL)	0.1, 0.1–0.6
Eosinophils (%)	2.00, 0.50–10.00
Basophils (× 10^3^ /µL)	0.00, 0.00–0.1
Basophils (%)	0.00, 0.00–1.00
UAS7 at first Omalizumab infusion	35.00, 18.00–42.00

Data are expressed as median and range

We divided the patient group in three subgroups based on the response to Omalizumab treatment:

- Early responders: UAS7 < 14 at T1
- Late responders: UAS7 < 20 at T2
- Non-responders: UAS7 unchanged at T2

The other group of controls was CIA patients: 11 patients (7 males and 4 females, median UAS7: 26, range 18–36) with Chronic Spontaneous Urticaria on anti-H1 therapy.

Informed consent was obtained from all patients and controls. The Investigations were carried out following the rules of the Declaration of Helsinki of 1975 revised in 2013. Approval of this study was obtained by the local Ethical Committee (Prot. PG/2021/153).

Blood was collected in citrate vacuum tubes (VenoSafe, Terumo Europe) made of polyethylene terephthalate, containing 0.109 M sodium citrate. Citrated blood was centrifuged at 2020 × g for 20 min at room temperature and the separated plasma was placed in polypropylene tubes. Plasma samples for analysis of Activated Partial Thromboplastin (aPTT) ratio, CWA, D-Dimer, Fibrinogen, thrombin generation and F_1+2_ assays were stored at − 80 °C after being kept in liquid nitrogen and were used for each patients and controls after thawing at 37 °C.

Activated Partial Thromboplastin (aPTT) ratio, CWA, D-Dimer and Fibrinogen were determined on an ACL TOP 550 CTS (Werfen, Barcelona, Spain) using the reagents SynthAsIL, D-Dimer HS, QFA HemosIL (Werfen, Barcelona, Spain), respectively. In particular, the method used for describing CWA was a turbidimetric method for clot detection. In this type of coagulometer, 0% absorbance defines the pre-coagulation phase then the absorbance (mAbs) increases after the initiation of clotting.

**Fig. 1:**
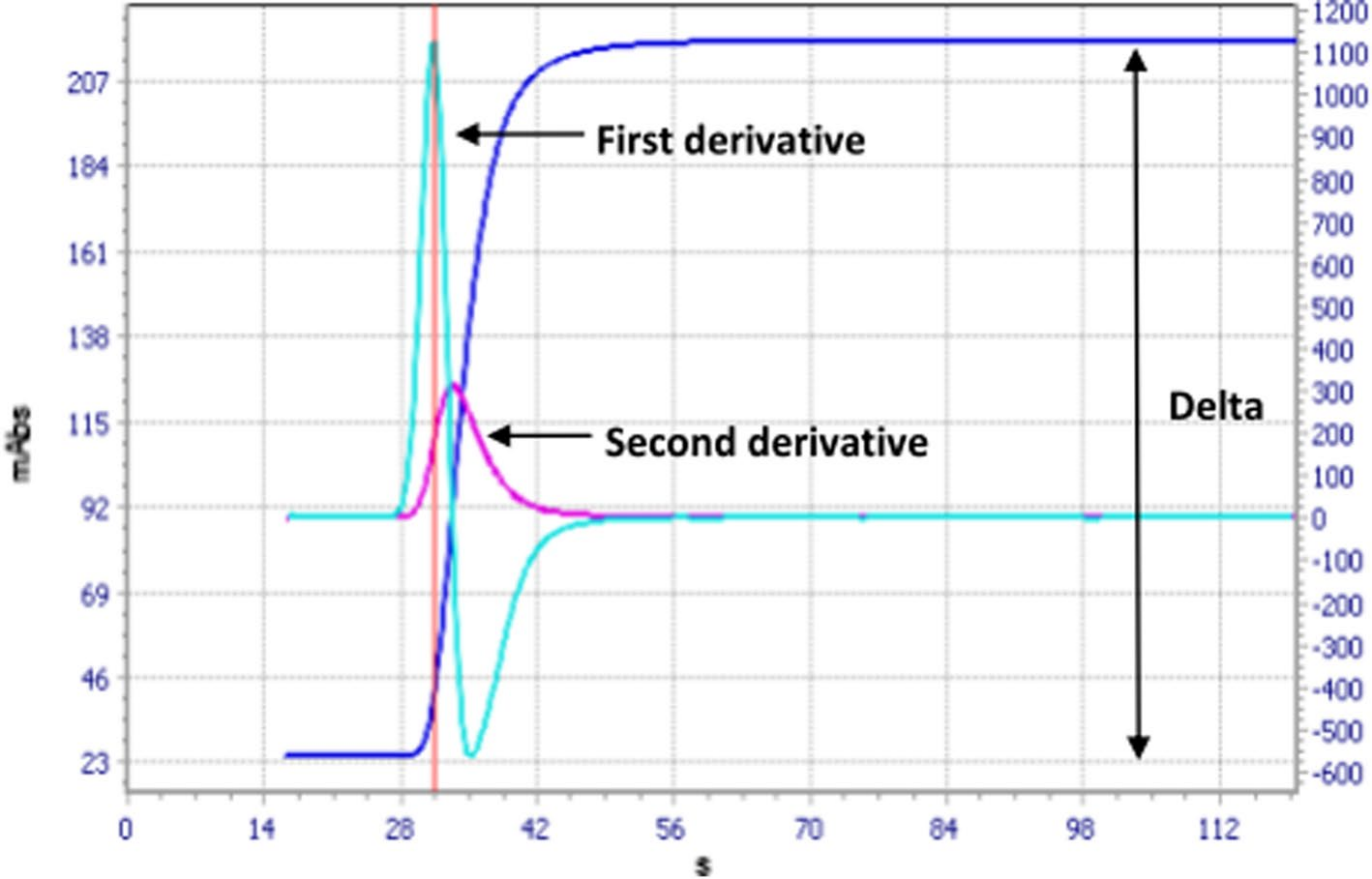
Example of first and second derivatives of aPTT and Delta aPTT in a healthy subject

CWA investigation has been previously described in other studies of ours [9, 10]. Briefly, the first derivative was the time at which maximum velocity of clot formation was reached (expressed as mAbs/s). The second derivative was the time at which maximum change in acceleration of clot formation was reached (expressed as mAbs/s^2^). Delta was the total change in optical density, that is, the maximum density of the clot (expressed as mAbs). CWA was derived from an implemented software dedicated to the ACL TOP 550 CTS [11]. This topic has been the subject of a communication by the subcommittees of the International Society of Thrombosis and Haemostasis (ISTH) aimed at the standardization of this procedure and the recommendations for its clinical application [12]. An example of CWA is shown in Fig. 1.

For F_1+2_ an enzyme immunoassay was used (Enzygnost F1 + 2, Siemens Healthcare S.r.l., Milano, Italy). It consists of an immuno-enzymatic method for the quantitative in vitro determination of the human F_1+2_ prothrombin fragment in plasma based on the sandwich principle with mouse monoclonal antibodies in microplate.

In 23 out of 47 patients with CSU Thrombin Generation (TG) was determined in Platelet Poor Plasma (PPP) using the Calibrated Automated Thrombogram (CAT) method (Diagnostica Stago, Asnières sur Seine France) [13]. Eighty µl of PPP were pipetted into the well of a microtiter plate together with 20 µl of PPP-Reagent + / − TM (with and without Thrombomodulin) (Thrombinoscope BV, Maastricht, The Netherlands), PPP-Reagent contains a mixture of Tissue Factor (5 pM final concentration) and synthetic phospholipids (4 µM final concentration), PPP-Reagent with Thrombomodulin contains a mixture of Phospholipids, Tissue Factor and Thrombomodulin (in the range of 4–6 nM). The reaction was started with 20 µl of a mixture composed of the fluorogenic thrombin substrate (Z-GlyGly-Arg-AMC, Thrombinoscope BV, 417 µM final concentration) and CaCl_2_ (15 mM final concentrations). The substrate is cleaved by the thrombin formed and liberates a fluorophore, which is converted to thrombin-equivalent concentrations (nM) using a reference curve [12]. To obtain a thrombin generation (TG) curve from the conversion of added fluorogenic substrate, thrombin concentrations are to be derived from the observed velocity of increase in fluorescence (dF/dt). Fluorescence was read in a Fluoroskan Ascent® reader (Thermo Fisher Scientific Corporation, Vantaa, Finland) and TG curves were calculated using the Thrombinoscope Software. Endogenous thrombin potential (ETP, area under the curve, nM*min was expressed as with/without thrombomodulin ratio. This ratio represents the resistance to the anticoagulant activity of TM and should be considered as an index of the procoagulant imbalance. The higher the ratio, the greater the procoagulant activity. The ETP is the area under the curve (AUC) that represents all the enzymatic activity of thrombin when it is activated. Therefore, it is the parameter that properly represents the coagulation phase. Finally, the platelet count and the Mean Platelet Volume were carried out using an automated cell counter (Yumizen HS2500, Horiba ABX SAS, Parc Euromédecine, Montpellier, France).

### Statistical analysis

As the variables examined were not normally distributed, data are presented as median and ranges. Accordingly, the Mann–Whitney test for independent data, the nonparametric analysis of variance (Kruskal–Wallis) and the Dunn's test (post hoc analysis) along with the Analysis of Variance for repeated measures, after log transformation, were carried out. MedCalc software (Version 17.7.2, Ostend, Belgium) was used for the statistical analysis of the data.

## Results

Considering the entire case series of 47 patients with CSU, a statistically significant increase in aPTT ratio, D-Dimer, F_1+2_, Platelet count and MPV were found at T0 in comparison with 53 HC. In contrast, the 2nd derivative of aPTT showed lower values than those of the HC. No differences were found between 1st derivative of aPTT, Delta aPTT and Fibrinogen. (Table 2).

**Table 2:** Mann–Whitney test for independent data

Parameters	CSU T0 Patients in T0 (before first Omalizumab infusion) *n* = 47	Healthy controls *n* = 53	*p*
aPTT ratio	1.19, 1.12–1.26	1.03, 1.01–1.06	< 0.0001
1st der aPTT (mAbs/s)	197.29, 173.75–220.84	218.52, 203.30–233.74	0.1229
2nd der aPTT MAX (mAbs/s^2^)	606.64, 523.69–689.59	746.31, 686.14–806.49	0.0065
aPTT Delta (mAbs)	75.39, 65.59–86.67	76.87, 70.56–83.75	0.8081
Fibrinogen (mg/dl)	317.98, 288.33–350.70	289.02, 272.60–306.41	0.0870
D-Dimer (ng/ml)	172.96, 125.45–238.47	101.94, 84.61–122.81	0.0041
F_1+2_ (pg/ml)	499.32, 412.02–605.12	141.32, 125.65–158.95	< 0.0001
Platelet count (10^9^/L)	244.00, 105.00–413.00	210.00, 154.00–394.00	0.0215
MPV (fL)	13.40, 7.80–19.60	11.20, 9.70–12-80	< 0.0001

All parameters studied in 47 CSU patients in T0 (before first Omalizumab infusion) and 53 healthy subjects. Data are expressed as median and range

As far as the group of 23 subjects with CSU a statistically significant difference was found in the ETP (nM*min) ratio (*p* = 0.0184; median 0.77, range 0.29–1.12). (Table 3).

**Table 3:** Mann–Whitney test for independent data

Parameters	CSU T0 Patients with CSU in T0 (before first Omalizumab infusion) *n* = 23	Healthy controls *n* = 53	*p*
aPTT ratio	1.11, 0.90–1.59	1.03, 0.81–1.26	0.0132
1st der aPTT (mAbs/s)	234.81, 81.94–452.70	207.66, 93.43–328.67	0.6228
2nd der aPTT MAX (mAbs/s^2^)	721.32, 203.28–1082.03	754.14, 224.12–1206.36	0.7905
aPTT Delta (mAbs)	86.00, 34.50–236.00	76.40, 45.90–181.70	0.1695
Fibrinogen (mg/dl)	354.00, 190.00–786.00	288.00, 182.00–429.00	0.0137
D-Dimer (ng/ml)	201.00, 27.00–1625.00	121.00, 23.00–280.00	0.0016
F_1+2_ (pg/ml)	484.00, 168.21–1157.97	133.00, 70.00–443.00	< 0.0001
Platelet count (10^9^/L)	244.00, 105.00–413.00	210.00, 154.00–394.00	0.0460
MPV (fL)	13.80, 7.80–19.50	11.10, 9.70–12.80	< 0.0001
LagTime (min) ratio	1.00, 0.77–1.45	0.94, 0.73–1.11	0.4477
ETP (nM*min) ratio	0.77, 0.29–1.12	0.67, 0.31–0.95	0.0184
Peak Height (nM) ratio	0.89, 0.56–1.10	0.85, 0.57–1.09	0.5452
Time to Peak (min) ratio	0.91, 0.60–1.28	0.87, 0.61–1.06	0.1795
Velocity Index (nM/min) ratio	0.98, 0.62–1.50	1.05, 0.66–1.51	0.2877

All parameters studied in 23 CSU patients in T0 (before first Omalizumab infusion) and 53 healthy subjects. Data are expressed as median and range

**Fig. 2:**
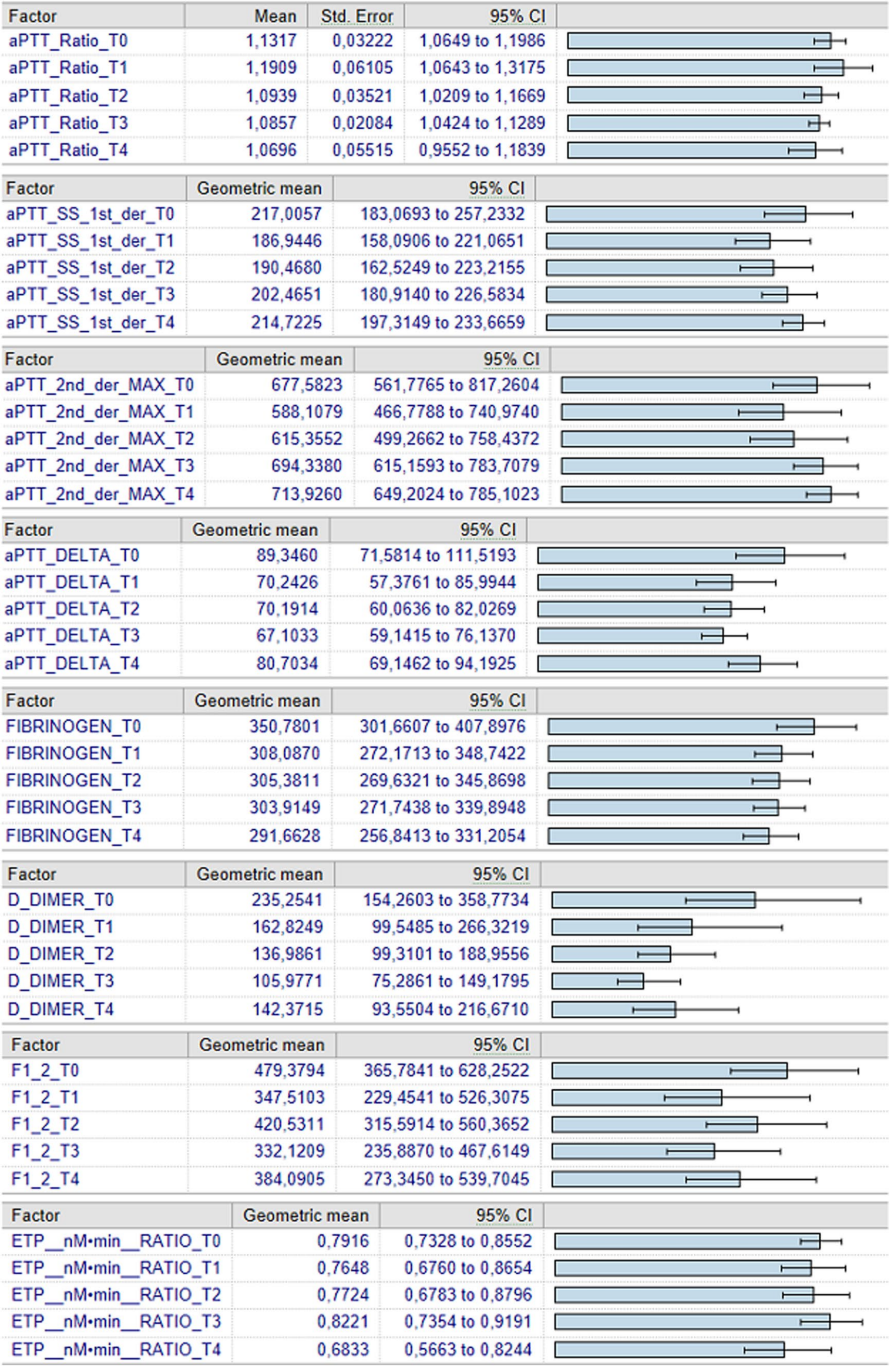
ANOVA for repeated measures, at T0, T1, T2, T3, T4. Data are expressed as mean and 95%CI

Analyzing through ANOVA for repeated measures, at T0, T1, T2, T3, T4, D-Dimer showed a significant difference between T0 and T3. No statistically significant difference was found among the other parameters at the different intervals examined (Fig. 2).

Only F_1+2_ showed a statistically significant difference between the 23 patients at T0 and the group with CSU on anti-histaminic drugs. No differences were found for the other parameters (Table 4).

**Table 4:** Mann–Whitney test for independent data

Parameters	CSU T0 Patients with CSU in T0 (before first Omalizumab infusion) *n* = 23	CIA Controls in antihistaminic *n* = 11	*p*
aPTT ratio	1.12, 0.90–1.59	1.07, 0.95–1.17	0.3291
1st der aPTT (mAbs/s)	234.81, 81.94–452.70	211.31, 148.84–353.36	0.7825
2nd der aPTT MAX (mAbs/s^2^)	718.37, 203.28–1082.03	720.83, 509.34–1207.11	0.7265
aPTT Delta (mAbs)	86.00, 34.50–236.00	68.80, 40.30–119.50	0.1014
Fibrinogen (mg/dl)	354.00, 190.00–786.00	267.00, 191.00–499.00	0.1564
D-Dimer (ng/ml)	201.00, 27.00–1625.00	118.00, 1.00–540.00	0.1791
F_1+2_ (pg/ml)	484.00, 168.00–1157.97	284.16, 132.20–693.50	**0.0343**
Platelet count (10^9^/L)	224.00, 105.00–413.00	220.00, 132.00–464.00	0.3112
MPV (fL)	14.30, 7.80–19.60	12.40, 8.20–19.20	0.3027
LagTime (min) ratio	1.00, 0.86–1.45	1.00, 0.90–1.08	0.5223
ETP (nM*min) ratioETP (nM*min) ratio	0.77, 0.60–1.12	0.73, 0.51–0.83	0.0993
Peak Height (nM) ratio	0.91, 0.63–1.10	0.85, 0.66–0.99	0.2887
Time to Peak (min) ratio	0.91, 0.73–1.28	0.89, 0.82–0.95	0.4159
Velocity Index (nM/min) ratio	0.98, 0.62–1.50	0.97, 0.80–1.48	0.6355

All parameters studied in patients with CSU at T0. (before first Omalizumab infusion) and in controls in antihistaminic (CIA). Data are expressed as median and range

Bold value indicates statistically significant (*p* < 0.05)

## Discussion

The pathophysiology of CSU is a further example of how the immune and the coagulative systems are each other linked. Eosinophils expose the tissue factor, which induce the activation of blood coagulation thus generating thrombin, which in turn is able to provoke vascular permeability on one hand and degranulation of mast cells with release of histamine on the other [14]. Increased levels of both F_1+2_ peptide, an indicator of blood coagulation activation, and D-dimer, a product of the fibrinolytic activity upon deposited fibrin, have been found in the course of urticaria [15, 16].

The concomitant increase in both F_1+2_ and D-Dimer clearly indicates fibrin deposition and dissolution. This mechanism may represent a low-grade consumption coagulopathy. In this study in the initial group of 47 patients we found significantly higher levels of F_1+2_ peptide and D-Dimer thus confirming the assumption exposed above. To reinforce this concept we also found two other interesting results. First, aPTT, a screening coagulative parameter, was found significantly prolonged when compared to controls. Even if this finding may be unexpected, it may indicate a mild consumption of coagulopathy. To support this statement, the detection of the aPTT derivatives indicates that the acceleration of the clot formation is decreased in the CSU patients. In fact, a significantly lower value of the 2nd derivative of aPTT was found in these patients. The behavior of the 1^st^ derivative of aPTT, which indicates the velocity of the clot formation, has been found to be similar but a significant statistical difference was not achieved. In other words, the abnormalities of CWA may explain the prolonged aPTT adding further support to the consumption coagulopathy hypothesis. Our findings are in contrast with those by Takeda et al. [17], who found a hypercoagulable state in CSU patients detecting, among several indicators of blood coagulation activation, higher values of the 1st and 2nd derivatives of aPTT so indicating both an increased velocity and acceleration of the clot formation. That study is the only published report, at least to our knowledge, on the behavior of CWA in CSU. To date, the reported alterations of a such hypercoagulable state in chronic urticaria have not been reported to be associated with an increased risk of thrombosis [18, 19]. However, in our study the concomitant presence of a prolonged aPTT along with a decrease in the CWA parameters further support the pathway which may be the basis of the coagulopathy in CSU. Another point to be addressed to the comprehension of this topic is the behavior of platelets in our patients. We found both an increase in platelet count and in Mean Platelet Volume (MPV). Our interpretation of these findings is that an increased platelet turnover is present in CSU thus further adding value to the hypothesis that a consumption coagulopathy does exist in CSU. MPV, in fact, is an indicator of peripheral platelet consumption since large platelets are delivered by the bone marrow in a compensatory way [20, 21]. Higher platelet count and MPV in CSU may be the effect of the inflammatory state of the disease: an ongoing and compensated platelet destruction-production process. On the other hand, an increased thrombin production, detected in this study by means of both ETG and F_1+2_ peptide, may be able to activate platelets so inducing a consumption which drives an increased production and turnover. Platelet aggregation during activation by thrombin can in turn accelerate thrombin generation mainly via platelet content release and platelet-derived extracellular vesicles formation [22]. The state of hypercoagulability in patients with CSU may therefore be the result of inflammation. However, it is a matter of debate whether the activation of coagulation has an initial role in CSU or is it secondary [23]; it is likely that the activation of coagulation participates in the pathogenesis of CSU even when the main mechanism is due to the presence of autoantibodies that cause the release of histamine [24].

In our case series, we observed that in the 23 patients prospectively followed during therapy with Omalizumab, the D-Dimer undergoes a decrease after the start of therapy with anti-IgE, which was significant when comparing the values at T0 with the values at T3. Only D-Dimer, among other biomarkers, has a lower concentration at T3 with respect to T0. None of the other clotting parameters show significant differences. Maybe our cohort is smaller to find substantial variations among Early Responders, Late Responders and Not Responders. The action of Omalizumab seems to be effective on clinical symptoms but not on blocking the subclinical state of hypercoagulability. A subclinical state of inflammation seems to persist, and it could explain the frequent relapses of disease after Omalizumab discontinuation. The activation of the coagulation system may be the biochemical spy of disease relapse. Indeed, it fits the idea of a clinical remission of the disease but a persistent biochemical activity. D-Dimer, Prothrombin fragment F_1+2_ and ETP ratio show a higher expression in CSU patients with respect to healthy controls. It fits the knowledge on the role of the clotting cascade and the fibrinolytic system in CSU. However, a possible explanation could be that fibrin deposition and dissolution could occur at the extravascular level as it happens in other inflammatory diseases [25]. These data support the idea that Chronic Spontaneous Urticaria should be considered as an immune-mediated chronic inflammatory disease resulting from multiple immunological factors activated by potential triggers [14].

This study has some limitations. First, the number of patients was small. However, we were able to assess all the parameters studied at four intervals. Second, the TG was directed only in the patients treated with Omalizumab because for the other patients this technique was not available.

## Conclusions

This study added to the knowledge of the pathophysiology of CSU the CWA which showed a decrease in the acceleration of the clot formation thus explaining the significantly prolonged aPTT in comparison with HC. This finding along with the hypercoagulable state detected by the increased levels of F_1+2_ peptide, D-Dimer and the TG may form a frame in which a low-grade DIC could be recognized. The accelerated turnover of platelets expressed by both an increase in platelet count and MPV further supports this pathway in CSU. Omalizumab was not able to normalize the coagulative parameters. D-Dimer was the only test which showed a decrease in the course of treatment with this anti-IgE monoclonal antibody. We therefore conclude stating that if it is true that Omalizumab is a successful treatment for almost CSU patients in terms of clinical outcomes, it does not affect a vicious circle consisting in a close relationship between the immune and the hemostatic systems. This assumption may be crucial in explaining why a relapse of the disease is easy to happen if the monoclonal antibodies are interrupted.
